# Comment on “Vitamin D Status in Migraine Patients: A Case-Control Study”

**DOI:** 10.1155/2014/635491

**Published:** 2014-03-31

**Authors:** Fevzi Nuri Aydin, Ibrahim Aydin, Mehmet Agilli

**Affiliations:** ^1^Department of Biochemistry, Sirnak Military Hospital, 73000 Sirnak, Turkey; ^2^Department of Biochemistry, Sarikamis Military Hospital, Sarikamis, 36500 Kars, Turkey; ^3^Department of Biochemistry, Agri Military Hospital, 04000 Agri, Turkey

We read with great interest the article by Alireza Zandifar et al. entitled* “Vitamin D status in migraine patients: a case-control study”* [1], in which they report no association between migraine and vitamin D status. However, we think that some points should be discussed.

In humans, vitamin D is derived mainly from the action of sunlight on the skin. Some factors alter the cutaneous production of vitamin D, such as age, melanin, sunscreens, covered dresses, drugs, time of the day, latitude, and glass. Sensible sun exposure (usually 5–10 minutes of exposure of the arms and legs or the hands, arms, and face, 2 or 3 times per week) protects body against vitamin D deficiency. Vitamin D deficiency is any serum 25(OH)D result less than 20 ng/mL. In this study, duration of sun exposure was mentioned as 120 min/day or ≥120 min/day. Although time is enough for synthesis, the mean level of plasma 25-hydroxyvitamin D (25(OH)D) was 13.55 ± 0.91 ng/mL in cases and 13.19 ± 1.19 ng/mL in controls. Women, Iranian people, who wore covered dresses were most likely to have low vitamin D levels [2]. These factors could have affected the results of the study. The frequency of female patients wearing covered dresses should have been mentioned.

UV-B radiation does not penetrate glass, so exposure to sunshine indoors through a window does not produce 25(OH)D [2]. The environmental conditions of the subjects in this study should be presented.

Several vitamin D metabolites are found in cerebral spinal fluid and have the ability to cross the blood-brain barrier. These vitamin D metabolites include 25(OH)D3, 1,25-dihydroxyvitamin D3 (1,25(OH)2D), and 24,25-dihydroxyvitamin D3. Addition of these metabolites in the study could show in more detail the relationship between migraine and vitamin D [3].

A 70-year-old person exposed to the same amount of sunlight as a 20-year-old person makes 25% of the vitamin D that the 20-year-old person can make. Older adults have a reduced level of 7-dehydrocholesterol, so they cannot synthesize 25(OH)D as well. Furthermore, their kidneys are less able to produce the active hormone, 1,25(OH)2D [2]. In this study, the distribution of age groups should be mentioned more clearly.

Melanin in the darker skin reduces the ability to produce 25(OH)D from sunlight exposure, because it absorbs the sunlight [2]. The frequency of dark-skinned subjects should be mentioned in the study.

Vitamin D is fat-soluble; it is sequestered in the body fat not allowing it to circulate. Moreover, those who have obesity cannot absorb vitamin D as readily [4]. Obesity is not among the exclusion criteria of the study; because of this, the results may have been influenced. Obesity should be stated in exclusion criteria in this study.

An inverse relationship between serum 25(OH)D and serum parathyroid hormone (PTH) is well known. When 25(OH)D levels are over 30 ng/mL, PTH concentration levels drop. If 25(OH)D concentrations are reduced to between 20 and 29 ng/mL, PTH concentration increases. In this study, PTH levels in case and control groups were not examined. Furthermore, hyperthyroidism and hypothyroidism can seriously affect the levels of vitamin D [5].

Recently, 25 experts from various medical disciplines drafted recommendations for vitamin D. As a result, serum is the recommended sample type. EDTA and other anticoagulants might have affected the measurements of 25(OH)D especially in immunoassays. Examples would be more accurate when using serum instead of plasma [6].

Consequently, explanation of these factors mentioned above could provide the readers with clearer information.
